# Maintenance of phosphate homeostasis and root development are coordinately regulated by MYB1, an R2R3-type MYB transcription factor in rice

**DOI:** 10.1093/jxb/erx174

**Published:** 2017-05-26

**Authors:** Mian Gu, Jun Zhang, Huanhuan Li, Daqian Meng, Ran Li, Xiaoli Dai, Shichao Wang, Wei Liu, Hongye Qu, Guohua xu

**Affiliations:** 1 State Key Laboratory of Crop Genetics and Germplasm Enhancement, Nanjing Agricultural University, Nanjing, China; 2 MOA Key Laboratory of Plant Nutrition and Fertilization in Lower-Middle Reaches of the Yangtze River, Nanjing, China

**Keywords:** Gibberellic acid biosynthesis, MYB transcription factor, *Oryza sativa* L, phosphorus, phosphate uptake, phosphate starvation response, root development

## Abstract

The adaptive responses of plants to phosphate (Pi) starvation stress are fine-tuned by an elaborate regulatory network. In this study, we identified and characterized a novel Pi starvation-responsive gene, *MYB1*, encoding an R2R3-type transcription factor in rice. *MYB1* was transcriptionally induced in leaf sheaths and old leaf blades. It was localized to the nucleus and expressed mainly in vascular tissues. Mutation of *MYB1* led to an increase in Pi uptake and accumulation, accompanied by altered expression of a subset of Pi transporters and several genes involved in Pi starvation signaling. Furthermore, MYB1 affected the elongation of the primary root in a Pi-dependent manner and lateral roots in a Pi-independent manner. Moreover, gibberellic acid (GA)-triggered lateral root elongation was largely suppressed in wild-type plants under Pi starvation conditions, whereas this suppression was partially rescued in *myb1* mutant lines, correlating with the up-regulation of a GA biosynthetic gene upon *MYB1* mutation. Taken together, the findings of this study highlight the role of MYB1 as a regulator involved in both Pi starvation signaling and GA biosynthesis. Such a co-regulator might have broad implications for the study of cross-talk between nutrient stress and other signaling pathways.

## Teaser

OsMYB1 affects phosphate homeostasis and root development by independent regulation of genes involved in phosphate starvation signaling and gibberellic acid biosynthesis.

## Introduction

Phosphorus (P) is an indispensable nutrient for plant growth and development, and serves as the constituent of many biologically important molecules (e.g. nucleic acids, phospholipids, and ATP) (Raghothama, 1999). However, the major form of P accessible to plant roots, namely inorganic orthophosphate (Pi), is often a significant limiting factor in natural ecosystems and agricultural production. This is due to the slow mobility of Pi in the soil and its fixation by cations and microorganisms (Plaxton and Tran, 2011). Plants have evolved a suite of developmental and metabolic responses to sustain their growth when exposed to Pi starvation stress, including modification of root system architecture (RSA), excretion of enzymes and organic acids, and formation of symbiotic associations with arbuscular mycorrhizal fungi (Smith *et al.*, 2011; Lambers and Plaxton, 2015; Li *et al.*, 2016). All these responses are fine-tuned by a complex gene-regulatory network (Chiou and Lin, 2011; López-Arredondo *et al.*, 2014; Liang *et al.*, 2014). In the past two decades, progress has been made to unravel this network. Genes of different molecular identities have been functionally characterized with regard to several aspects of Pi starvation responses. These genes have been pinned to the Pi starvation signaling network through the elucidation of their molecular and genetic interactions (Gu *et al.*, 2016; Luan *et al.*, 2016). However, much work remains to be done to get closer to a comprehensive understanding of this network and then the potential applications of the expanded knowledge (Tian *et al.*, 2012; Gu *et al.*, 2016).

Transcription factors (TFs), along with other regulatory genes involved in Pi signaling, affect Pi homeostasis through direct or indirect regulation of the Pi transporters (PTs) at different levels (Gu *et al.*, 2016). Transcriptional activation/repression represents an early and important step of gene expression regulation, which is largely dependent on TFs and has been recognized as an important level of regulation in plants encountering Pi starvation stress (Bustos *et al.*, 2010; Secco *et al.*, 2013). To date, an increasing number of TFs belonging to several families have been reported to be involved in plant Pi starvation responses in both model plants and crops (Yi *et al.*, 2005; Chen *et al.*, 2007; Devaiah *et al.*, 2007a, b; Camacho-Cristóbal *et al.*, 2008; Devaiah *et al.*, 2009; Chen *et al.*, 2009; Liu *et al.*, 2009; Dai *et al.*, 2012; Baek *et al.*, 2013; Shen *et al.*, 2013; Ramaiah *et al.*, 2014; Singh *et al.*, 2014; Wang *et al.*, 2014a, b; Yang *et al.*, 2014; Chen & Schmidt, 2015; Dai *et al.*, 2016; Ruan *et al.*, 2017). Among these TFs, members of the MYB family have been most intensively studied. A group of MYB TFs designated as PHR (Phosphate Starvation Response), which recognize and bind to a non-perfect palindromic *cis*-element P1BS (PHR1-Binding Sequence), have been defined as central regulators of Pi starvation signaling and found to be conserved in diverse plant species. In *Arabidopsis thaliana*, PHR1 and PHL1 (PHR1-Like1) work in combination to control the transcriptional expression of a considerable proportion of PSI (Phosphate Starvation-induced) genes (Rubio *et al.*, 2001; Bustos *et al.*, 2010). Among the genes acting downstream of AtPHR1, *microRNA399* (*miR399*), which post-transcriptionally targets a ubiquitin-conjugating E2 enzyme (*PHO2*, *PHOSPHATE2*), has been shown to be important for maintaining plant Pi homeostasis (Aung *et al.*, 2006; Bari *et al.*, 2006). Two close homologs of *AtPHR1/AtPHL1* (*AtPHL2* and *AtPHL3*), which are induced by Pi starvation at both the transcriptional and the protein level, have been reported to play redundant roles in the transcriptional control of Pi starvation signaling (Sun *et al.*, 2016). In rice (*Oryza sativa*), OsPHR1/OsPHR2/OsPHR3 function in a highly conserved manner to that found in Arabidopsis PHR1/PHLs (Zhou *et al.*, 2008; Guo *et al.*, 2015). Very recently, another rice homolog, *OsPHR4*, has been found to be a direct target of OsPHR1/OsPHR2/OsPHR3 and to play a similar role to its counterparts (Ruan *et al.*, 2017). AtPHR1 and OsPHR2 are barely responsive to Pi starvation at the transcriptional level, whereas their activities are post-translationally governed by proteins of the SPX (SYG1/PHO81/XPR1) family (Puga *et al.*, 2014; Wang *et al.*, 2014). AtSPX1 and OsSPX1 function as negative regulators of Pi starvation signaling via physical interaction with AtPHR1 and OsPHR2, respectively (Puga *et al.*, 2014; Wang *et al.*, 2014). In addition to PHRs/PHLs, which are 1R-type MYB TFs, several R2R3-type MYB members, namely OsMYB2P-1, OsMYB4P, AtMYB2, and AtMYB62, have also been reported to be involved in Pi starvation signaling. All these R2R3-type MYB TFs are transcriptionally up-regulated by Pi starvation in roots and/or shoot tissues and affect Pi homeostasis by regulating PSI genes (Devaiah *et al.*, 2009; Dai *et al.*, 2012; Baek *et al.*, 2013; Yang *et al.*, 2014).

Eight out of nine known phytohormones, namely auxin, cytokinin, abscisic acid, ethylene, jasmonic acid, brassinosteroid, strigolactone, and gibberellic acid (GA), have been implicated in Pi starvation signaling or other pathways triggered by Pi starvation (Hammond and White, 2008, 2011; Singh *et al.*, 2014; Khan *et al.*, 2016). Among the reported R2R3-type MYB members involved in Pi starvation signaling, *AtMYB62* shows a unique expression pattern, which is specifically induced in leaves, and it simultaneously modulates GA biosynthesis and flowering by suppressing the expression of genes responsible for these two processes (Devaiah *et al.*, 2009).

In the present study, a novel PSI gene encoding an R2R3-type MYB TF, *MYB1*, was identified in rice. We show that MYB1 affects Pi homeostasis by negatively regulating the expression of PT genes. MYB1 was also found to be involved in Pi- and/or GA-dependent root development. This study pins another node to the Pi starvation signaling network, and presents an entry point by which to dissect the molecular mechanisms underlying the cross-talk between Pi starvation signaling and phytohormone pathways in crops.

## Materials and methods

### Plant materials and growth conditions

The Nipponbare cultivar of rice (*Oryza sativa* L. ssp. *japonica*) was used for most experiments in this study. The T-DNA insertion line *osphr2* (Line ID RMD_04Z11NL88) was obtained from the Rice Mutant Database (RMD) maintained by the National Center of Plant Gene Research (Wuhan) at Huazhong Agricultural University; its genetic background is *japonica* cv. Zhonghua11. Hydroponic experiments were performed using normal rice culture solution containing 1.425 mM NH_4_NO_3_, 0.2 mM NaH_2_PO_4_, 0.513 mM K_2_SO_4_, 0.998 mM CaCl_2_, 1.643 mM MgSO_4_, 0.009 mM MnCl_2_, 0.075 mM (NH_4_)_6_Mo_7_O_24_, 0.019 mM H_3_BO_3_, 0.155 mM CuSO_4_, 0.02 mM Fe-EDTA, and 0.152 mM ZnSO_4_. Rice plants were grown in growth chambers with a 12 h light/12 h dark photoperiod and a day/night temperature of ~28/20 ℃ after germination. Seedlings were treated with ~0.25–0.5 strength nutrient solution (described above) until the fifth leaf blade had just emerged. Subsequently, the seedlings were treated with high phosphate (HP; 200 μM Pi), low phosphate (LP; 10 μM Pi) or no phosphate (NP; 0 μM Pi) until the seventh leaf blade was fully expanded, and then evaluated for phenotype or sampled.

### RNA extraction, cDNA preparation, and RT-qPCR

Total RNA was extracted from various tissues using TRIzol reagent (Invitrogen). Genomic DNA elimination and reverse transcription were performed using the PrimeScript RT reagent Kit with gDNA Eraser (TaKaRa Biotechnology, Dalian, China). Reverse transcription–quantitative PCR (RT-qPCR) was performed with the SYBR Premix Ex Taq^TM^ II (Perfect Real Time) Kit (TaKaRa Biotechnology) on the StepOnePlus Real-Time PCR System (Applied Biosystems). Relative expression levels were normalized to that of *OsActin1* (LOC_Os03g50885) and presented as 2^−△CT^.

### Subcellular localization analysis by agroinfiltration of tobacco leaves

The full-length open reading frame (ORF) of *MYB1* without the stop codon was first ligated to an intermediate vector, pSAT6A-EGFP-N1. The *MYB1::GFP* fusion construct as well as *GFP* alone were then subcloned into the expression vector pRCS2-ocs-nptII with the rare cutter PI-PspI. The plasmids were both transformed into *Agrobacterium tumefaciens* (EHA105). The cells were harvested by centrifugation and resuspended in a solution containing 10 mM MES, 10 mM MgCl_2_, and 200 μM acetosyringone (pH 5.7) at an optical density (600 nm) of 0.1. Cell suspensions were incubated at room temperature for 1–4 h and then infiltrated into the leaves of *Nicotiana benthamiana* by using a needle-free syringe. Green fluorescent protein (GFP) fluorescence in the transformed leaves was imaged using a confocal laser scanning microscope (Leica SP8) after 48–72 h.

### Transactivation assay using the yeast two-hybrid system

The full-length ORF of *OsMYB1* was amplified by PCR and cloned into pBD-GAL4 Cam with *Eco*RI and *Sma*I, to produce an in-frame fusion with the yeast GAL4 DNA-binding domain. The *OsMYB1-pBD-GAL4* construct was transformed into YRG-2 cells by using the lithium acetate-mediated method, and transformants were selected on synthetic dextrose (SD) medium lacking tryptophan (W). Yeast transformants from SD/-W were then streaked on to solid SD/-W or SD/-W/-Histidine (H) medium for observation of growth.

### Construction of expression vectors for tissue localization analysis and *OsMYB1* mutation, and generation of transgenic plants

For tissue localization analysis, the GUSPlus reporter gene and the NOS terminator were introduced into pCAMBIA1300 via *Kpn*I/*Sac*I and *Sac*I/*Eco*RI, respectively, resulting in a new expression vector, pCAMBIA1300-GN. A 2149 bp fragment upstream of the translation start codon of *OsMYB1* was amplified and fused upstream of the GUSPlus reporter gene via *Bam*HI/*Kpn*I. For gene mutation with the CRISPR-Cas9 system, three different spacers residing in exons were selected from the library provided by Miao *et al.* (2013). These spacers were ligated to the intermediate vector pOs-sgRNA via *Bsa*I and then introduced into pH-Ubi-cas9-7 through the use of GATEWAY technology.

The constructs were transformed into mature embryos developed from seeds of wild-type (WT) rice plants (cv. Nipponbare) via *A. tumefaciens*-mediated transformation as previously described (Jia *et al.*, 2011).

### Phosphate uptake assay

WT plants and *myb1* mutants were grown in Pi-sufficient or Pi-deficient solution until the seventh leaf blades were fully expanded. The roots of the plants were incubated in a pretreatment solution (2 mM MES and 0.5 mM CaCl_2_, pH 5.5) before moving them into a 1 l uptake solution (nutrient solution plus 100 μM NaH_2_PO_4_, pH 5.5) containing [^32^P]orthophosphate (8 µCi·l^−1^) and incubated for 6, 10, and 24 h. After three washes with double-distilled H_2_O, samples were moved into ice-cold desorption solution (2 mM MES, 0.5 mM CaCl_2_, 100 μM NaH_2_PO_4_, pH 5.5). The samples were blot dried, weighed, and placed in scintillation vials with 1 ml perchloric acid and 0.5 ml hydrogen peroxide. The scintillation vials were then placed in an oven overnight at 65 ℃. Subsequently, 0.2 ml of supernatant was transferred to a 5 ml vial and 3 ml scintillation cocktail (ULTIMA GOLD^TM^ LLT; PerkinElmer) was added to each vial, and radioactivity was measured with a scintillation counter (Beckman Coulter).

### Measurement of soluble Pi concentration

For the measurement of soluble Pi concentration in the plant, ~0.5 g fresh samples were used following the method previously described by Zhou *et al.* (2008). Briefly, frozen samples were homogenized in 1 ml 10% (w/v) perchloric acid, using an ice-cold mortar and pestle. The homogenate was then diluted 10-fold with 5% (w/v) perchloric acid and placed on ice for 30 min. After centrifugation at 10000 *g* for 10 min at 4 ℃, the supernatant was used for Pi measurement by using the molybdenum blue method: 0.4% (w/v) ammonium molybdate dissolved in 0.5 M H_2_SO_4_ (solution A) was mixed with 10% ascorbic acid (solution B) at a ratio of A:B=6:1. A 2 ml aliquot of this working solution was added to 1 ml of the sample solution and incubated in a water bath at 42 ℃ for 20 min. After being cooled, the absorbance at 820 nm was measured using a SpectraMax M5 multi-detection microplate reader system (Molecular Devices, Sunnyvale, CA, USA).

### Root traits analysis

Rice seeds were surface sterilized and washed with deionized water three times, and then germinated in the dark. When the first incomplete leaf was fully developed and the primary roots were ~2 cm in length, the seedlings were transferred to a hydroponic system and supplied with (200 μM Pi) or without (0 μM Pi) phosphate. After the third leaf blades of the seedlings were fully expanded, the roots were cut off from the seedlings for further observation. All the roots were scanned at 600 dpi by using a Perfection V700 Photo scanner (Epson).

## Results

### Cloning and characterization of *MYB1*

**Fig. 1.**
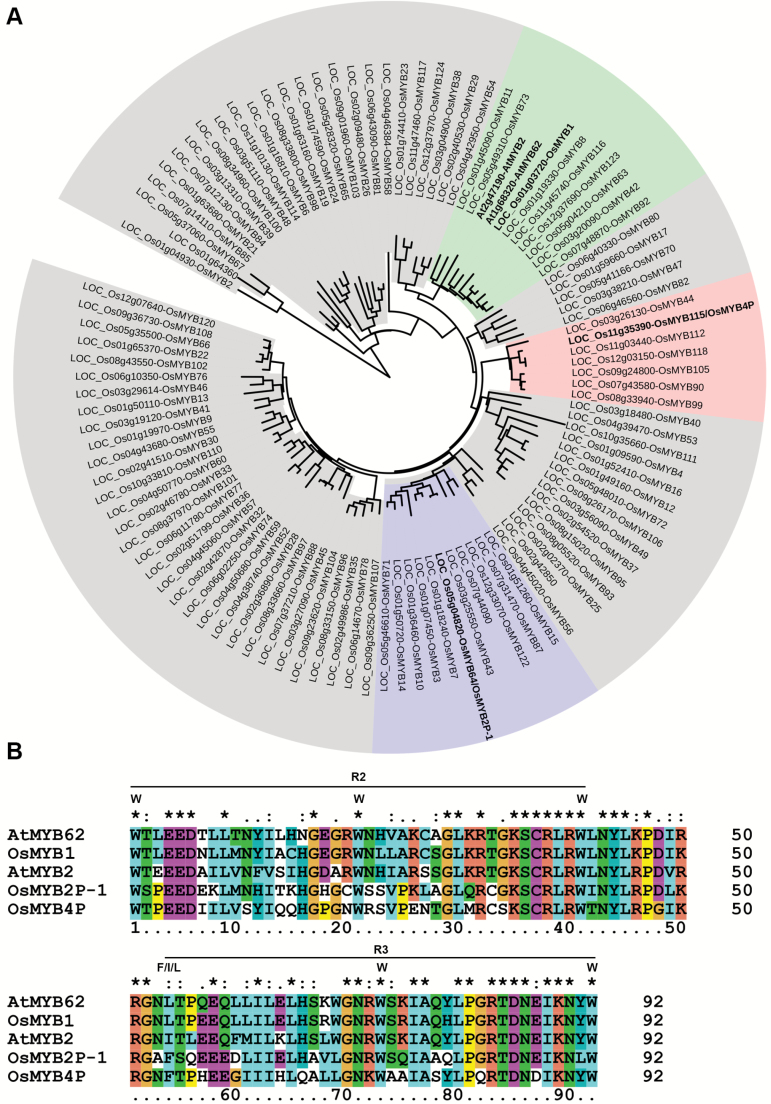
Phylogenetic and amino acid sequence analysis of OsMYB1. (A) Phylogenetic analysis of the R2R3-type MYB transcription factors from rice and selected counterparts in *A. thaliana*. The gene names are based on nomenclature suggested by Gray *et al.* (2009). The phylogenetic tree was generated using the MYB domain of the candidates. The gene names of four reported R2R3-type MYB transcription factors involved in Pi starvation signaling and OsMYB1 are highlighted in bold, and the clades to which these five members belong are highlighted in red, green, and blue. (B) Alignment of the MYB domains of OsMYB1 and reported R2R3-type MYB transcription factors involved in Pi starvation signaling.

The amino acid sequences of the reported R2R3-type MYB TFs involved in Pi starvation signaling and those of all the homologs in rice were retrieved for phylogenetic analysis (http://planttfdb.cbi.pku.edu.cn/index.php?sp=Osj). The results showed that the R2R3-type MYB TFs were clustered into different clades, and LOC_Os01g03720 (Os01g0128000; designated as *MYB1*) was most closely related to AtMYB62 (Fig. 1A). Protein domain analysis by the Pfam database (http://pfam.xfam.org/) revealed that the deduced protein sequence of LOC_Os01g03720 bears two MYB repeats at its N terminus, which show high sequence identity to the reported R2R3-type MYB TFs (Fig. 1B).

**Fig. 2.**
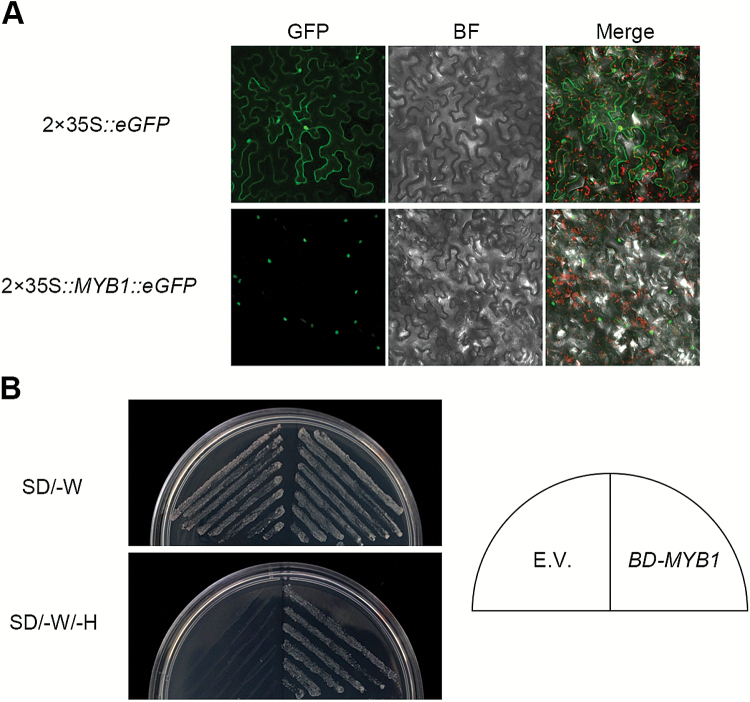
Subcellular localization and transcriptional activity analysis of OsMYB1. (A) Subcellular localization analysis of the OsMYB1::GFP fusion protein by *A. tumefaciens*-mediated infiltration of tobacco (*N. benthamiana*) leaf epidermal cells. BF, bright field. (B) Transcriptional activity analysis of OsMYB1 protein. Transformants with pBD-GAL4 [empty vector (E.V.) serving as a negative control] and pBD-GAL4-OsMYB1 were streaked on to synthetic dextrose (SD) medium lacking tryptophan (W) (upper panel) or both tryptophan and histidine (H) (lower panel).

To investigate the subcellular localization of *MYB1*, the *GFP* reporter gene was fused to the 3ʹ terminus of its ORF. The fusion was driven by the 35S cauliflower mosaic virus promoter and transformed into the leaf epidermal cells of *N. benthamiana* by *A. tumefaciens*-mediated infiltration. GFP, which served as a positive control, was detectable throughout the cell, whereas the MYB1::GFP fusion protein was confined to the nucleus (Fig. 2A). In addition, a yeast GAL4 TF-based system was used for determining the transcriptional activity of MYB1. The full-length ORF of *MYB1* was fused to the GAL4 DNA-binding domain (BD). Transformation of the *GAL4-BD::MYB1* fusion, but not of *GAL4-BD* alone, restored yeast growth on synthetic dextrose medium lacking tryptophan and histidine (SD/-W/-H; Fig. 2B), indicating that MYB1 possesses transcription-activating activity. Together, these findings suggest that MYB1 might be a nucleus-localized R2R3-type MYB TF.

### *MYB1* is responsive to Pi starvation in shoot tissues

**Fig. 3.**
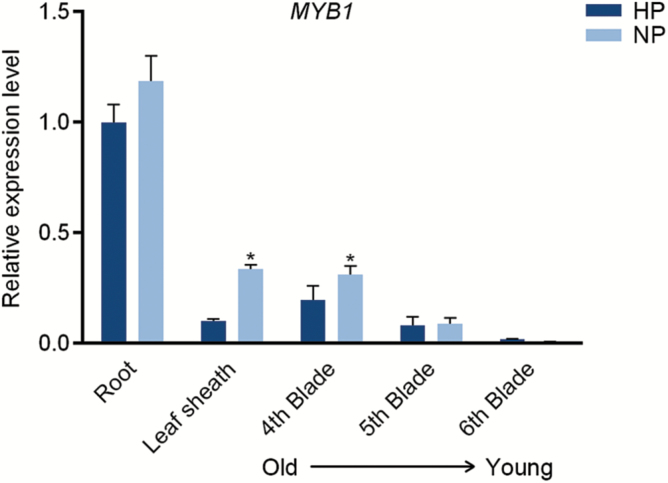
Expression of *OsMYB1* in response to Pi starvation. Rice seeds were germinated in deionized H_2_O and supplied with (HP) or without (NP) Pi. Roots, leaf sheaths, and three leaf blades (the fourth to the sixth) were collected from seedlings. RT-qPCR analysis was performed using the rice housekeeping gene *OsActin1* (LOC_Os03g50885) as an internal control. Values presented are the means±SD of three biological replicates. * *P*<0.05 (Student’s *t*-test).

Given the high sequence identity shared by *MYB1* and *AtMYB62*, which is induced by Pi starvation specifically in leaves (Devaiah *et al.*, 2009), the transcriptional expression of *MYB1* in response to Pi starvation was determined by RT-qPCR analysis. *MYB1* was expressed in all the tissues examined, and showed the greatest abundance in roots (Fig. 3). Similar to findings in Arabidopsis (Devaiah *et al.*, 2009), the expression of *MYB1* was not significantly up-regulated in response to Pi starvation in roots, while induction by Pi starvation was evident in leaf sheaths and older (the fourth) leaf blades (Fig. 3). To explore the potential link between MYB1 and the central Pi starvation signaling pathway in rice, the expression of *MYB1* was also tested in *phr2* mutant plants. The results showed that *MYB1* was up-regulated in the shoots of Pi-replete *phr2* mutants (see Supplementary Fig. S1 at JXB online), suggesting that *MYB1* expression is suppressed by PHR2. In addition, no P1BS *cis*-element was found to be present in the promoter region of *MYB1*, indicating that *MYB1* might not be a direct target of PHR2. These data are consistent with the established notion that the control of transcriptional repression responses by PHR TF is indirect (Bustos *et al.*, 2010).

### *MYB1* shows preferential tissue localization

**Fig. 4.**
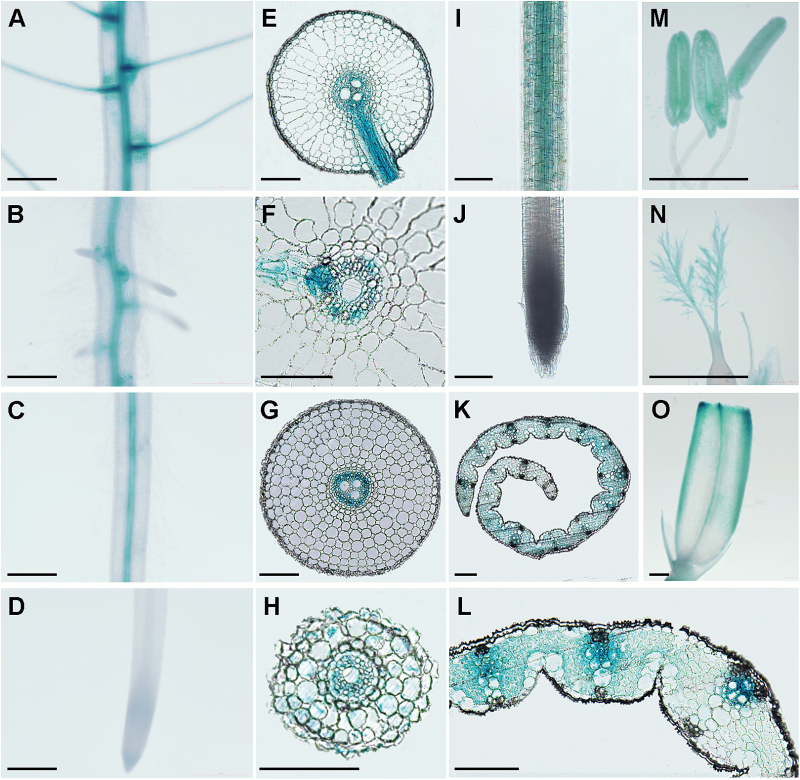
Histochemical staining for GUS activity in transgenic rice plants expressing a *P*_*OsMYB1*_*::GUS* fusion. Plants were grown hydroponically and supplied with sufficient Pi. (A–D) Different zones of primary root. (E–G) Cross-sections of the root segment shown in A (E), B (F), and C (G). (H) Cross-section of the basal part of lateral root. (I) Basal part of lateral root. (J) Root tip of lateral root. (K) Cross-section of leaf sheath. (L) Cross-section of leaf blade. (M) Stamens. (N) Pistil. (O) Lemma and palea. Scale bars in A–D and M–O indicate 100 µm; bars in E–L indicate 500 µm.

To determine the tissue localization of *MYB1*, a 2149 bp genomic sequence upstream of its translation start codon was amplified and fused to the *GUS* reporter gene. Transgenic rice plants were generated with the *P*_*MYB1*_*::GUS* construct. In primary roots, GUS activity was mainly detectable in the stele and lateral root primordia, as well as in emerged lateral root (Fig. 4A–G). Notably, GUS staining was absent in the root cap and meristem zone of the primary root tip (Fig. 4D). A similar distribution of GUS activity was observed in lateral roots, except that a moderate level of expression was also present in the outer layers of cells (Fig. 4H–J). In leaf sheaths and leaf blades, GUS activity was detectable throughout all cell types, with the strongest expression level in the vascular bundles of leaf blades (Fig. 4K, L). We also examined GUS activity in reproductive tissues. The results showed that *MYB1* was expressed in anther, stigma, lemma, and palea (Fig. 4M–O).

### MYB1 negatively affects Pi uptake and accumulation

**Fig. 5.**
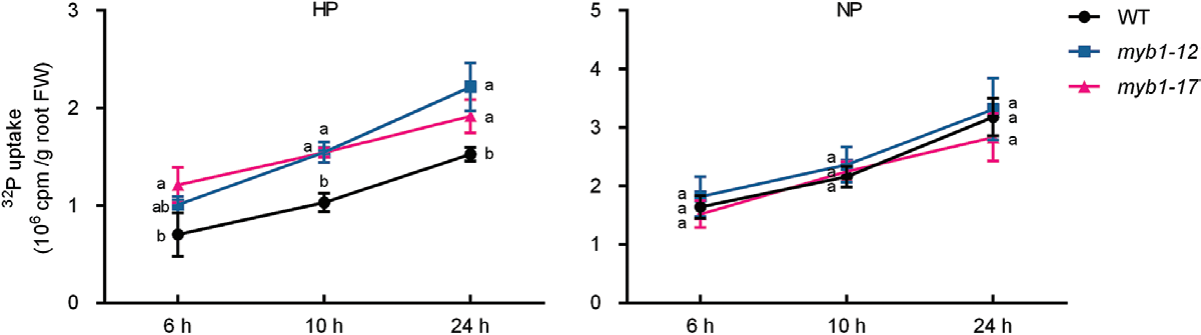
Uptake of Pi in *myb1* mutants and WT plants. The *myb1* mutants and WT plants were grown in Pi-sufficient (HP, 200 µM Pi) and Pi-deficient (NP, 0 µM Pi) solutions until the seventh leaf blades were fully expanded, and were then placed in a hydroponic system containing 100 µM Pi labeled with radioactive ^32^P. The Pi uptake of the plants was monitored over a 24 h period. Left panel, Pi uptake in plants grown in HP solution; right panel, Pi uptake in plants grown in LP solution. Values presented are means±SD (*n*=3). Different letters indicate significant differences (*P*<0.05, Duncan’s test).

**Fig. 6.**
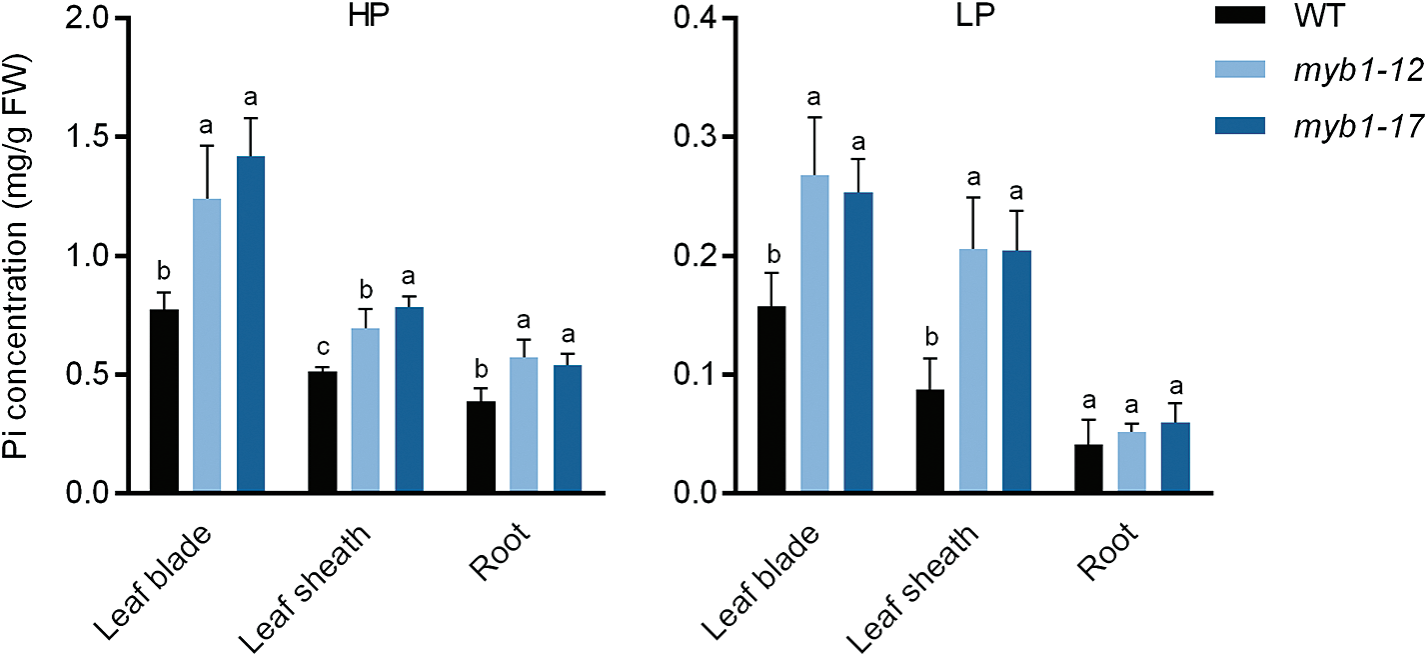
Concentration of Pi in leaf tissues and roots of WT and *myb1* mutant plants. Four-leaf-old seedlings were grown hydroponically and supplied with 200 μM (HP) or 10 μM (LP) Pi until the seventh leaf blades were fully expanded. The Pi concentration was measured in plants grown under HP (left panel) and LP (right panel) conditions. Leaf blades, leaf sheaths, and roots were collected for measurement. Error bars indicate SD (*n*=6). Different letters indicate significant differences (*P*<0.05, Duncan’s test). FW, fresh weight.

The physiological roles and molecular regulation of Arabidopsis *MYB62* have been investigated in overexpression plants (Devaiah *et al.*, 2009). Our attempts to generate *MYB1* overexpression lines in either rice or Arabidopsis were unsuccessful. Therefore, to study the potential role of *MYB1* in the maintenance of Pi homeostasis, mutant lines of *MYB1* were generated by means of CRISPR-Cas9 technology (Miao *et al.*, 2013). Eight independent mutant lines (derived from three spacers) were identified and selected for preliminary analysis of Pi accumulation. All eight lines showed a higher Pi concentration in leaf blades compared with that of WT plants (data not shown). Two of the eight mutant lines (*myb1-12* and *myb1-17*) were selected for further analysis (see Supplementary Fig. S2). Four-leaf-old plants of the mutant lines and the WT plants were grown in a hydroponic system until the seventh leaf blades were fully expanded. HP (200 μM) and NP (0 μM) treatments were used for monitoring Pi uptake, and HP and a LP (10 μM) treatments were applied for measuring Pi accumulation by the rice plants. A significant increase in the short-term Pi uptake rate of *myb1* mutant roots was observed under the HP condition, but not under the NP condition, compared with the uptake by WT roots (Fig. 5). In addition, the Pi concentration was increased in leaf blades, leaf sheaths, and roots of *myb1* mutants under the HP condition compared with that in the corresponding WT tissues (Fig. 6). Under the LP condition, an increase in Pi concentration in *myb1* mutants was observed only in leaf tissues and not in roots (Fig. 6).

### MYB1 regulates the expression of genes involved in Pi transport and signaling

**Fig. 7.**
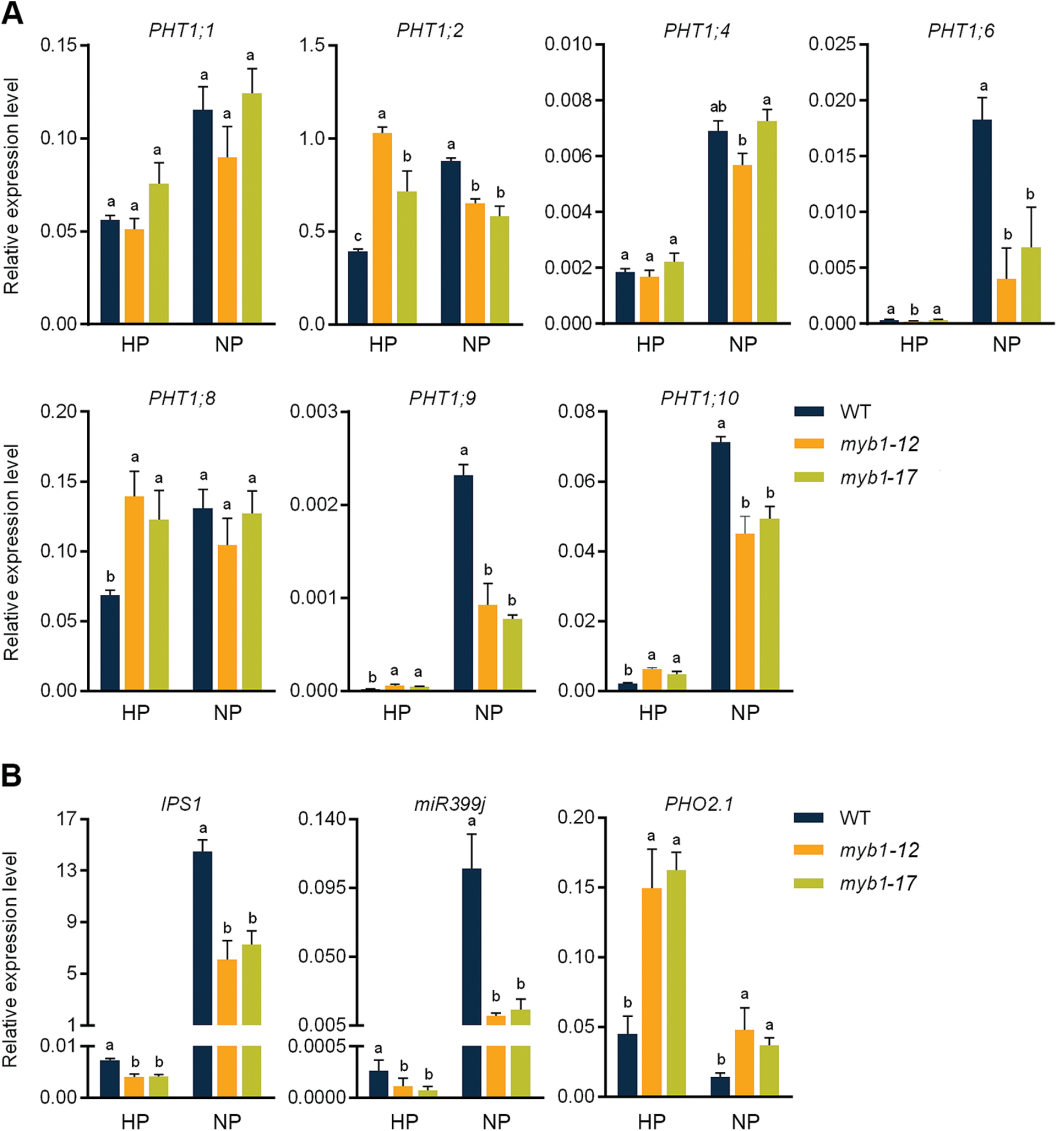
Effect of *OsMYB1* mutation on the expression of genes involved in Pi transport and signaling. (A) RT-qPCR analysis of PHT1 genes in *myb1* mutants and WT plants. (B) RT-qPCR analysis of a subset of genes involved Pi signaling in *myb1* mutant and WT plants. Seeds of the mutant lines and WT plants were germinated in deionized H_2_O and supplied with (HP, 200 µM Pi) or without (NP, 0 µM Pi) Pi. The rice housekeeping gene *OsActin1* (LOC_Os03g50885) was used as an internal control. Values are presented as the means±SD of three biological replicates. Different letters indicate significant differences within a treatment (*P*<0.05, Duncan’s test).

To investigate the effect of MYB1 on Pi signaling, the expression of a subset of Pi starvation-responsive genes was examined. As expected, all the genes responded to Pi starvation normally in the WT plants, that is, being induced or suppressed (*PHO2.1*) by Pi starvation (Fig. 7). Under Pi sufficient conditions, two major PHT1 members, *PHT1;2* and *PHT1;8*, as well as *PHT1;9* and *PHT1;10*, were significantly up-regulated in *myb1* mutant lines (Fig. 7). Notably, *PHT1;2* and *PHT1;8* showed comparable or even higher levels of expression in the *myb1* mutant lines under Pi sufficient conditions compared with the Pi starvation condition (Fig. 7), unlike the findings in WT plants, in which expression of these genes was greater under Pi starvation. Interestingly, under the Pi starvation condition, the expression of several PHT1 members (*PHT1;2*, *PHT1;6*, *PHT1;9*, and *PHT1;10*) and some PSI genes was suppressed in the *myb1* mutants relative to the WT plants, whereas the Pi starvation-suppressed isoform of the miR399 target, *PHO2.1*, was up-regulated; this latter finding was probably due to the down-regulation of *miR399* (Fig. 7).

### MYB1 affects the elongation of primary roots and lateral roots in response to exogenous GA and/or Pi starvation

**Fig. 8.**
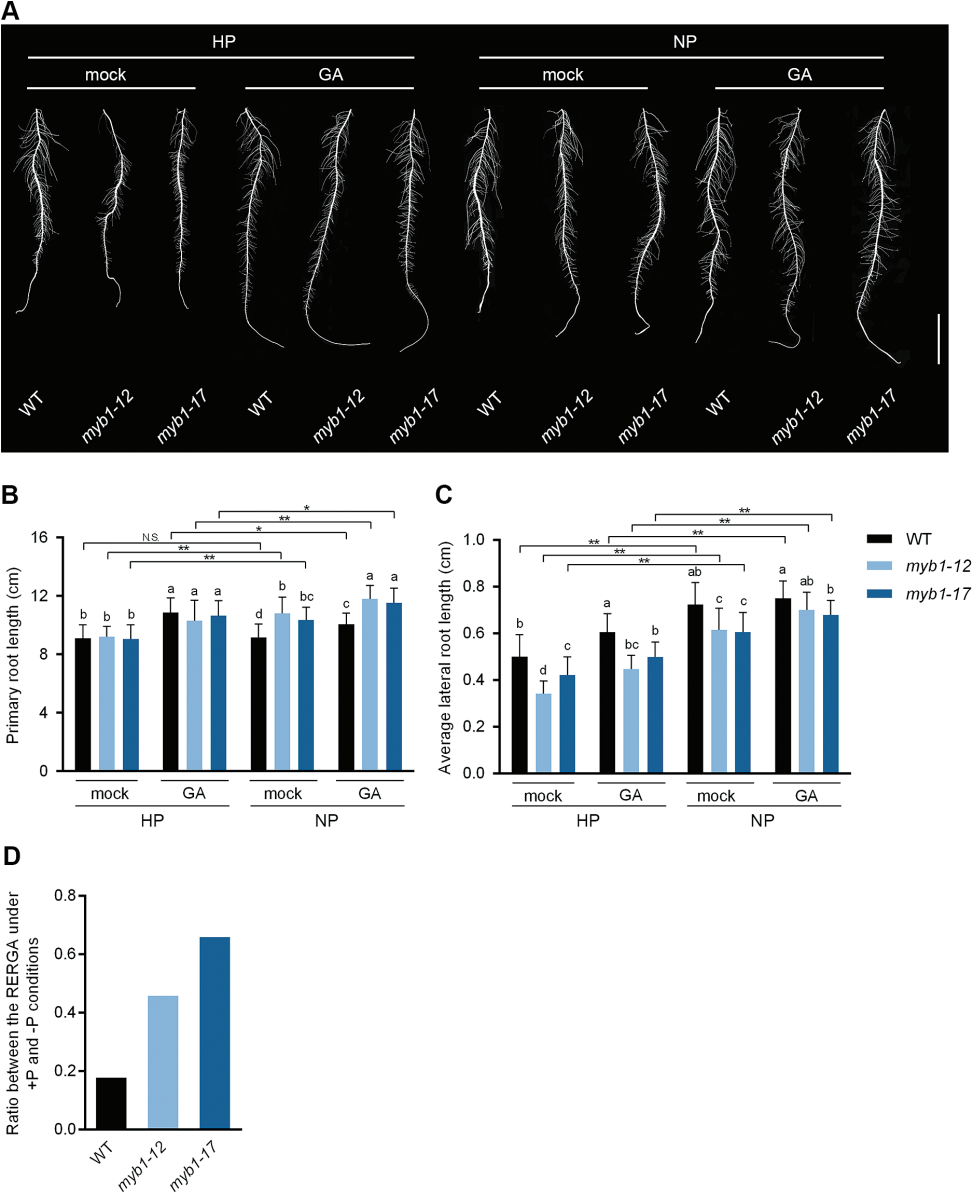
Root trait analysis of *myb1* mutants and WT plants grown in different Pi regimes. Uniformly germinated seeds of *myb1* mutants and WT plants were grown hydroponically with (HP, 200 μ Μ Pi) or without (NP, 0 μ Μ Pi) Pi for 5 days. (A) Overview of the primary root architecture of *myb1* mutants and WT plants. The roots of each seedling were cut off and scanned at 600 dpi. Shown are representatives of at least 15 seedlings. Scale bar=2 cm. (B) Primary root length. (C) Average lateral root length. (D) The ratio of the RERGA [root elongation ratio in response to exogenous GA; (average lateral root length under GA treatment – average lateral root length under control condition) ÷ average lateral root length under control condition] under the Pi starvation condition and the RERGA under the Pi sufficient condition. Values presented are means±SD (*n*=15–20). Different letters indicate significant differences within the HP or the NP condition (*P*<0.05, Duncan’s test). Asterisks indicate significant differences across conditions, between the two indicated values: * *P*<0.05, ** *P*<0.01, N.S., not significant (Student’s *t*-test).

The role of GA in the Pi starvation response in Arabidopsis has been documented, and AtMYB62 is known to affect plant development by regulating the expression of GA-biosynthetic genes (Jiang *et al.*, 2007; Devaiah *et al.*, 2009). Given that MYB1 shares the highest sequence identity with AtMYB62 (Fig. 1) and displays a similar role in Pi starvation signaling (Fig. 7A), it would be interesting to know whether MYB1 is involved in GA metabolism. Unexpectedly, no visible GA-related phenotype (e.g. the length of the second leaf sheath; Tong *et al.*, 2014) was observed in the shoots (see Supplementary Fig. S3). Therefore, a combination of exogenous GA and Pi starvation treatments was applied to the roots of *myb1* mutants and WT plants in an attempt to study the effect of the mutation on root development. It has been reported that GA has a positive effect on the elongation of primary roots in rice (Tong *et al.*, 2014). A consistent response of the primary roots to GA, namely an increase in the primary root length, was observed in both WT plants and *myb1* mutants irrespective of Pi status (Fig. 8A, B). Furthermore, mutation of *MYB1* led to a significant increase in primary root length under the Pi starvation condition, independent of GA supply (Fig. 8A, B).

The average length of lateral roots was significantly increased by Pi starvation in both WT plants and *myb1* mutants, irrespective of whether GA was supplied (Fig. 8A, C). Under the Pi sufficient condition, the average length of lateral roots significantly increased in response to exogenous GA in both WT plants and *myb1* mutants (Fig. 8A, C). Under the Pi starvation condition, the length of lateral roots of WT plants treated with GA was comparable to that of plants mock-treated with solvent, suggesting that the potential effect of GA on the elongation of lateral roots was blocked by Pi starvation stress (Fig. 8A, C). However, the response of lateral roots to exogenous GA (GA-triggered elongation) under the Pi starvation condition was largely retained in the *myb1* mutants (Fig. 8A, C); this response could also be reflected by the increased ratio of the RERGA [root elongation ratio in response to exogenous GA, calculated as (average lateral root length under GA treatment – average lateral root length under control condition) ÷ average lateral root length under control condition] under the Pi starvation condition and the RERGA under the Pi sufficient condition (Fig. 8D).

### MYB1 alters the expression of GA biosynthetic genes

**Fig. 9.**
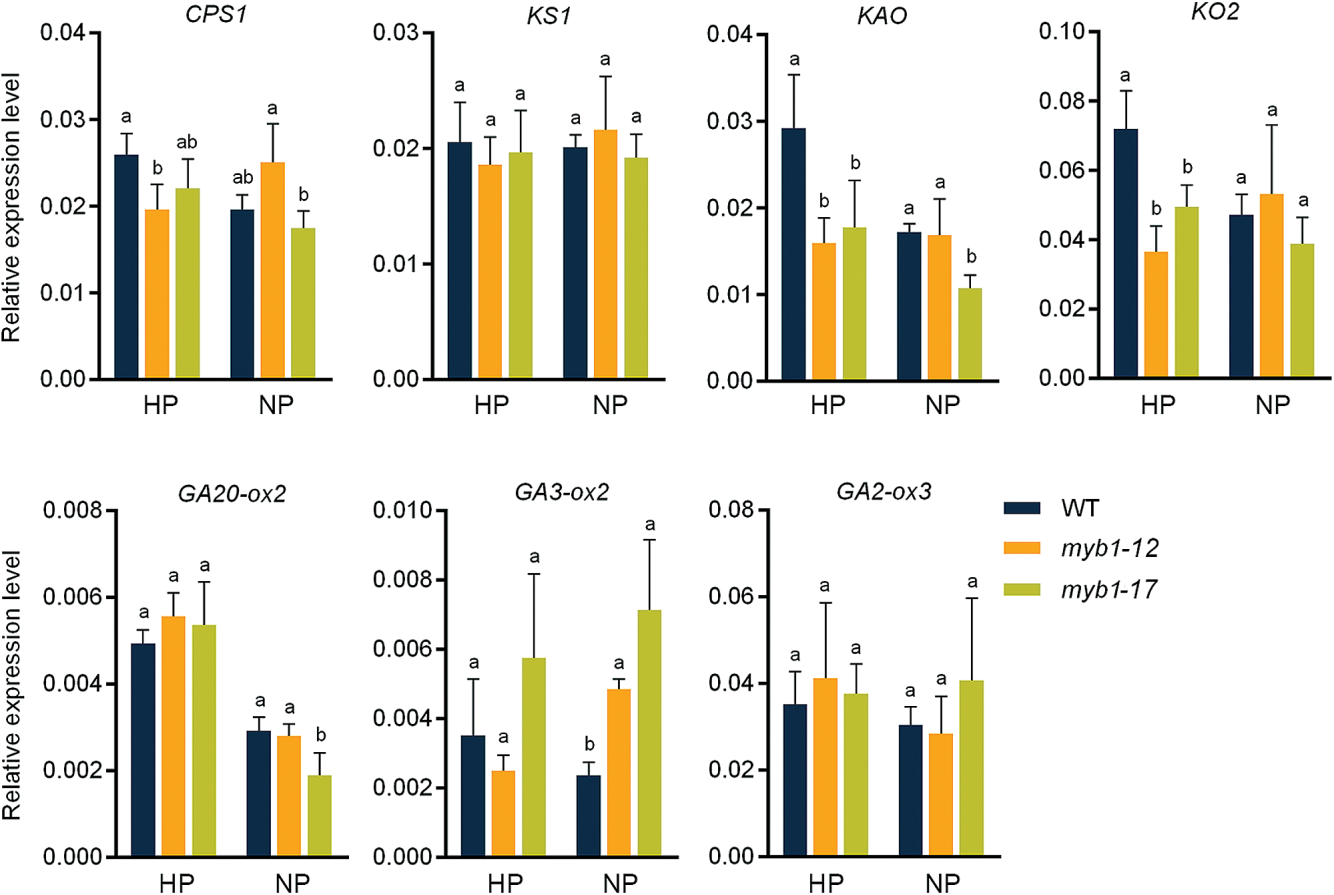
Effect of *OsMYB1* mutation on the genes involved in gibberellic acid biosynthesis. The same templates as that in Fig. 7 were used for RT-qPCR analysis. The rice housekeeping gene *OsActin1* (LOC_Os03g50885) was used as an internal control. The values presented are means of three biological replicates. Error bars indicate SD. Different lowercase letters indicate significant differences within a treatment (HP or NP) at *P*<0.05 (Duncan’s test).

We also tested the expression of the genes responsible for GA biosynthesis and signaling in the *myb1* mutant lines. Unlike Arabidopsis AtMYB62, which serves as a negative regulator of GA biosynthetic genes (Devaiah *et al.*, 2009), MYB1 positively regulates the expression of GA biosynthetic genes under Pi sufficient conditions, as evidenced by the fact that several genes responsible for GA biosynthesis (*CPS1*, *KO2*, and *KAO*) were down-regulated in the Pi-replete *myb1* mutants compared with their levels of expression in WT plants (Fig. 9). In contrast, another gene that functions at a later step of the GA biosynthesis pathway, *GA3ox-2*, was significantly up-regulated in *myb1* mutants under the Pi starvation condition (Fig. 9), suggesting that there was an increase in GA biosynthesis in Pi-starved *myb1* mutants.

## Discussion

Recent studies on the TFs involved in Pi starvation signaling have greatly increased our knowledge on the regulatory mechanisms that operate at different levels (Gu *et al.*, 2016). However, much remains to be done in order to achieve a comprehensive understanding of the Pi starvation signaling network, as well as its cross-talk with other pathways such as phytohormone signaling cascades. The present study provides an additional line of evidence that a single TF could be responsible for both the maintenance of plant Pi homeostasis and the phytohormone-induced modification of RSA, providing an entry point for dissecting the cross-talk between nutritional cues and the phytohormone signaling pathway.

### MYB1 is a negative regulator of Pi uptake

As the closest homolog of MYB1, AtMYB62 has been demonstrated to be a negative regulator of PSI genes. Two major PHT1 genes in Arabidopsis, *AtPHT1;1* and *AtPHT1;4*, were found to be down-regulated in *AtMYB62*-overexpressing plants under Pi-deficient conditions (Shin *et al.*, 2004; Devaiah *et al.*, 2009; Ayadi *et al.*, 2015). However, the Pi uptake of *AtMYB62*-overexpressing plants is enhanced under Pi sufficient conditions, owing to altered RSA (Devaiah *et al.*, 2009). In the present study, we found that *PHT1;2* and *PHT1;8* were significantly up-regulated by the mutation of *MYB1* under Pi sufficient conditions (Fig. 7), in accordance with the increase in Pi uptake and accumulation in *myb1* mutants (Figs 5, 6). These findings suggest that MYB1 might function as a negative regulator of PSI genes and Pi uptake when there is ample external Pi. Among the rice PHT1 genes, *PHT1;2* and *PHT1;8*, together with *PHT1;1*, showed considerably higher expression levels in Pi-replete roots compared with other family members (Fig. 7; Secco *et al.*, 2013), indicating their fundamental roles in Pi uptake and translocation under Pi sufficient conditions. Indeed, *PHT1;2* and *PHT1;8* have been demonstrated to be two major PT genes for Pi uptake and translocation in rice, which are regulated at both transcriptional and post-translational levels (Ai *et al.*, 2009; Liu *et al.*, 2010; Chen *et al.*, 2011, 2015; Jia *et al.*, 2011). Furthermore, our previous work and Liu *et al.* (2010) have shown that overexpression of either of these two PHT1 genes leads to increased Pi uptake and/or accumulation (Liu *et al.*, 2010; Jia *et al.*, 2011), yet no obvious symptoms of Pi toxicity, such as those observed in *PHT1;2*- and *PHT1;8*- overexpressing rice plants, were observed in *myb1* mutants (data not shown; Ai *et al.*, 2009; Jia *et al.*, 2011; Chen *et al.*, 2015). Nevertheless, the enhanced expression of *PHT1;2* and *PHT1;8*, as well as that of *PHT1;9* and *PHT1;10*, in Pi-replete *myb1* mutants correlates well with the increased Pi uptake and accumulation observed in the mutants (Figs 5–7) and could be the direct cause. Moreover, a considerable number of reported rice Pi over-accumulators show constitutive Pi starvation responses under Pi sufficient conditions (Hu *et al.*, 2011; Dai *et al.*, 2012; Guo *et al.*, 2015; Dai *et al.*, 2016). No similar responses were found in the *myb1* mutants, since the PSI marker genes were not induced or even down-regulated when the Pi supply was sufficient (Fig. 7B). This indicates that the regulatory effect of MYB1 on Pi signaling might not be universal to most PSI genes, which is the case in the PHR TFs.

In contrast to the results obtained under Pi sufficient conditions, when the Pi supply was limited, neither PHT1 members nor genes involved in Pi starvation signaling were found to be up-regulated by *MYB1* mutation; on the contrary, several PHT1 members and PSI marker genes were down-regulated in the roots of Pi-starved *myb1* plants (Fig. 7). It is noteworthy that in the *myb1* mutants, the transcriptional responses of *PHT1;2* and *PHT1;8* to Pi starvation were absent (*PHT1;8*) or even opposite (*PHT1;2*) to those of the WT plants (Fig. 7A). This finding suggests that the regulatory effect of MYB1 on *PHT1;2* and *PHT1;8* is dependent on the Pi supply. Given that *MYB1* is constitutively expressed in roots irrespective of the Pi regime (Fig. 3), a Pi-dependent post-translational event regulating the activity of MYB1 (transcriptional activation/repression of downstream PSI genes) could be expected. Taking these findings together, it could be postulated that MYB1 functions as a negative regulator of the PSI genes under Pi sufficient conditions, and as and a positive regulator of the same genes under Pi starvation conditions. Alternatively, it cannot be excluded that regulation by MYB1 (i.e. repression of the expression of PSI genes) mainly takes place when there is ample external Pi, and the down-regulation of the PSI genes observed under Pi starvation conditions is an indirect effect of improved Pi accumulation in *myb1* mutants (Figs 6, 7). On the other hand, *MYB1* is preferentially expressed in the vascular tissues of roots (Fig. 4), reminiscent of the spatial distribution of *PHT1;2* (Ai *et al.*, 2009). *PHT1;8* is also highly expressed in root cylinder cells (Jia *et al.*, 2011). These data further strengthen the hypothesis that MYB1 functions as a negative regulator of Pi uptake by suppressing the expression of Pi transporter genes.

### MYB1 regulates root development in a GA- and/or Pi-dependent manner

In Arabidopsis, a model plant with a tap root system, attenuation of primary root elongation is a well-known response to Pi starvation stress (Svistoonoff *et al.*, 2007; Pérez-Torres *et al.*, 2008; Gruber *et al.*, 2013; Müller *et al.*, 2015). However, it has been reported that this response is absent in a small but considerable proportion of Arabidopsis accessions (Reymond *et al.*, 2006). The primary roots of rice plants, which have a fibrous root system, also show different responses to Pi starvation. In Nipponbare, the first sequenced *japonica* rice cultivar, the primary roots are not very responsive to Pi starvation (Goff *et al.*, 2002; Shimizu *et al.*, 2004; Zhou *et al.*, 2008; Hu *et al.*, 2011). Consistent with these previously reported results, we found that the primary root length of Pi-starved WT plants was comparable to that of Pi-replete WT plants. However, Pi starvation led to a significant increase in the primary root length of the *myb1* mutants (Fig. 8A, B). This is similar to reported findings in two rice Pi over-accumulators, namely *PHR2* overexpression plants and *pho2* mutants (Zhou *et al.*, 2008; Hu *et al.*, 2011). These two Pi over-accumulators and the *myb1* mutants all display enhanced Pi uptake and accumulation, probably due to an increased abundance of Pi transporters. Given that the alteration of RSA in response to Pi starvation is mainly affected by local Pi signaling (Péret *et al*., 2002), much still has to be done to dissect the potential role of Pi transporters in local Pi sensing. Furthermore, under Pi starvation conditions, the primary root length of the *myb1* mutants was longer than that of the WT plants owing to the positive response of *myb1* roots to Pi starvation, which was not affected by exogenous GA (Fig. 8A, B). In Arabidopsis, exogenous GA can partially counteract the inhibition of primary root elongation caused by Pi starvation stress (Jiang *et al.*, 2007), whereas the effect of GA on primary root elongation is negligible when the Pi supply is sufficient (Jiang *et al.*, 2007; Ramaiah *et al.*, 2014). By contrast, the promotion of primary root elongation by GA is more evident in rice, irrespective of the Pi regime, although the greatest increase is no more than 20% (Tong *et al.*, 2014; Fig. 8A, B). The mechanism underlying the conservation and divergence of the primary root response to exogenous GA between the two model plants will require further investigation.

GA affects other aspects of the PSI modification of RSA in addition to primary root elongation (Jiang *et al.*, 2007; Ramaiah *et al.*, 2014). In Arabidopsis, a physiological level of GA is required for the elongation of root hairs in response to Pi starvation, and the Pi starvation-triggered increase in lateral root intensity and the development of secondary lateral roots are both partially inhibited by exogenous GA (Jiang *et al.*, 2007). Moreover, exogenous GA can promote the elongation of Arabidopsis lateral roots under normal Pi conditions (Ramaiah *et al.*, 2014). In the present study, GA was also found to be a positive regulator of lateral root elongation under Pi sufficient conditions in both WT plants and *myb1* mutants (Fig. 8A, C). Interestingly, under the Pi starvation condition, the positive response of lateral roots to GA was observed only in *myb1* mutants and not in WT plants (Fig. 8A, C). These results suggest that the effect of GA on the elongation of lateral roots was blocked by Pi starvation stress in WT plants. This is consistent with findings in Arabidopsis, in which Pi starvation antagonizes the effect of exogenous GA (Jiang *et al.*, 2007). Thus, we postulate that the conserved mechanism in monocots (rice) and dicots (Arabidopsis) might be attributed to the impairment of GA biosynthesis under Pi starvation conditions, and the up-regulation of a GA biosynthetic gene, *GA3-ox2*, in Pi-starved *myb1* mutants could lead to enhanced GA level and thus lateral root length. Notably, although Pi starvation suppresses GA biosynthesis, Pi starvation and GA both positively regulate the elongation of lateral roots (Fig. 8A, C), indicating that the regulation of lateral root elongation by Pi starvation stress and GA might occur through independent pathways.

It has been demonstrated that GA does not regulate PSI changes in Pi uptake efficiency or the expression of PSI genes (Jiang *et al.*, 2007), whereas MYB1 is involved in both Pi starvation signaling (Fig. 7) and the GA biosynthesis pathway (Fig. 9). These results demonstrate that MYB1 represents a link in the cross-talk between nutrient signaling and the phytohormone signaling pathway.

## Supplementary data

Supplementary data are available at *JXB* online.

Fig. S1. Expression of *OsMYB1* in *osphr2* mutants.

Fig. S2. Identification of *myb1* mutant lines.

Fig. S3. The phenotype of *myb1* mutants and wild-type plants in response to phosphate starvation and exogenous gibberellic acid.

Table S1. Primers used for constructs for subcellular localization and yeast two-hybrid assay.

Table S2. Primers used for constructs for generating transgenic plants.

Table S3. Primers used for RT-qPCR analysis.

## Supplementary Material

Supplementary Figures S1-S3 and Tables S1-S3
