# Supplementary material for: Maintenance of phosphate homeostasis and root development are coordinately regulated by MYB1, an R2R3-type MYB transcription factor in rice
Source: J Exp Bot. 2017 May 26;68(13):3603–15. doi: 10.1093/jxb/erx174 (PMC5853628; doi:10.1093/jxb/erx174)
Supplement: Supplementary Figures S1-S3 and Tables S1-S3 [file erx174_suppl_supplementary_figures_s1_s3_tables_s1_s3.pdf]

Supplementary Data

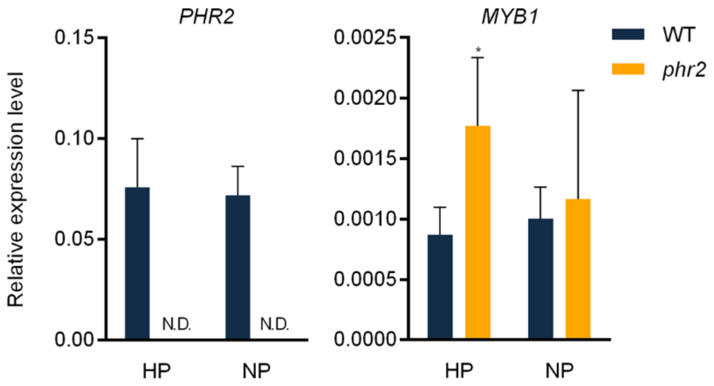

Fig. S1. Expression of *OsMYB1* in *osphr2* mutants. Rice seeds were germinated in deionized H_2O and supplied with (HP) or without (NP) phosphate. RT-qPCR analysis was performed using the rice housekeeping gene *OsActin1* (LOC_Os03g50885) as an internal control. The values presented are means of three biological replicates. Error bars indicate SD. * $P < 0.05$ (Student's *t*-test).

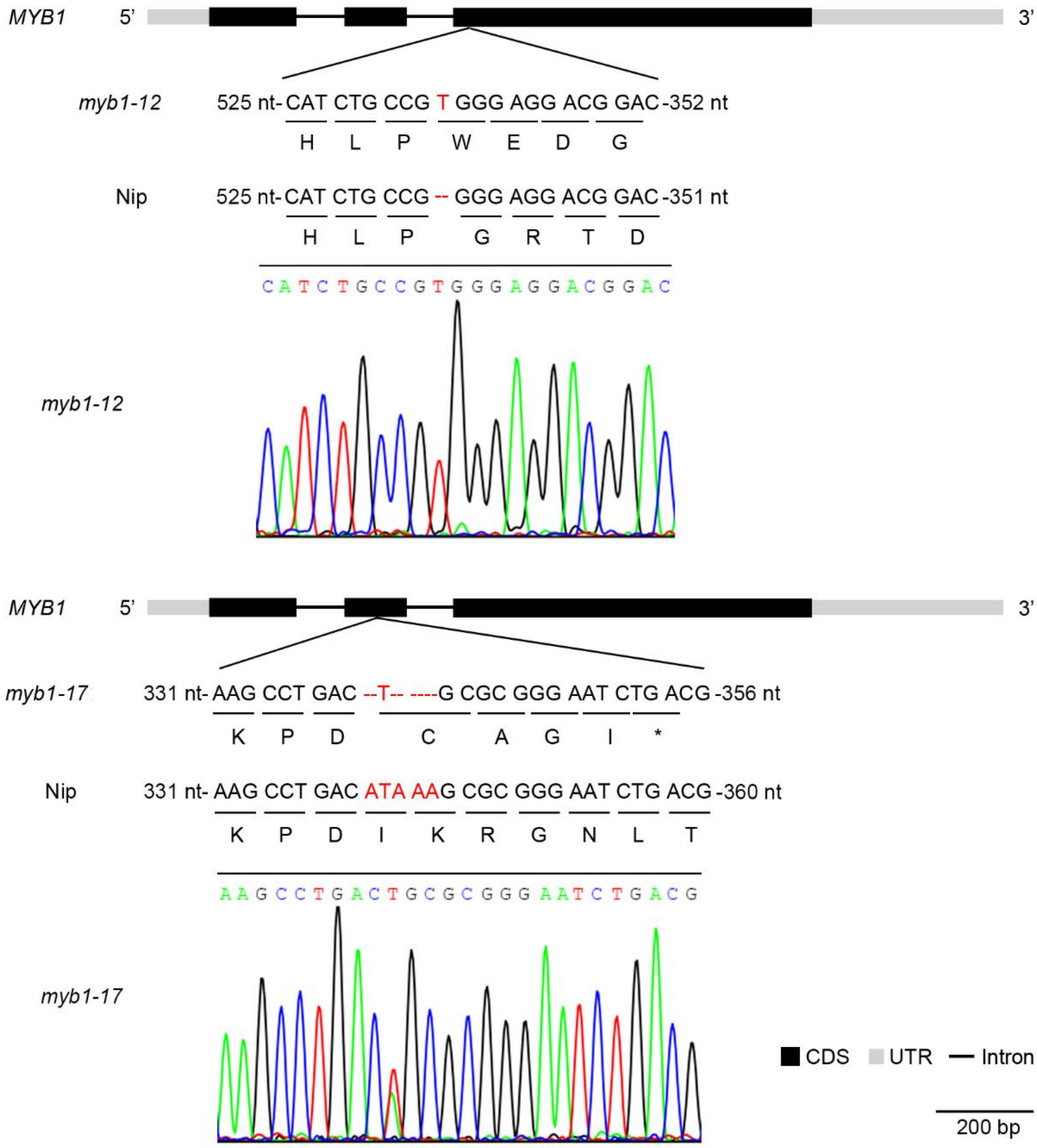

Fig. S2. Identification of *myb1* mutant lines. The gene structure of *OsMYB1* along with the mutation sites of three independent lines are present in each panel (upper, middle and lower). The coding sequences (CDS), the untranslated regions (UTR) and the introns are indicated by black boxes, grey boxes and black bars, respectively. The red letters indicate newly generated sequences by the CRISPR-Cas9 system. The letters underlying the nucleotide sequences are amino acid sequences.

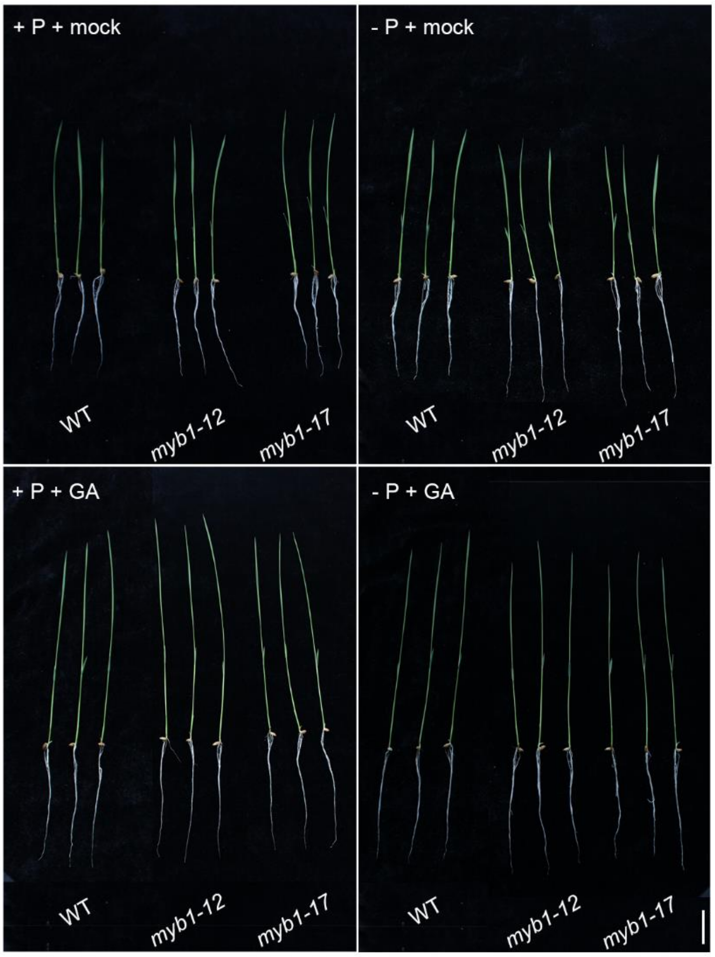

Fig. S3. The phenotype of *myb1* mutants and wild-type plants in response to phosphate starvation and exogenous gibberellic acid. The seedlings used for root trait study in Figure 8 were photographed for phenotypic observation. Scale bar = 2 cm.

Table S1. Primers used for constructs for subcellular localization and yeast-two-hybrid

Primer name	Sequence(5' to 3')	Note	Construct
**For subcellular localization**			
MYB-GFP_F	TTagatctATGTCGCAGAGGAGAGTTGCA	BglII	pSAT6A-EGFP-N1
MYB-GFP_R	TTcccgggGATTCCTCCTCCCCACTCGC	SmaI	
**For Y2H**			
MYB-pBD_F	TTgaattcATGTCGCAGAGGAGAGTTGCA	EcoRI	pBD-GAL4 Cam
MYB-pBD_R	TTcccgggGATTCCTCCTCCCCACTCGC	SmaI	

Table S2. Primers used for constructs for generating transgenic plants

Primer name	Sequence(5' to 3')	Note	Construct
**For promoter-*GUS* expression constructs of *MYB108***			
MYB-pro_F	TTggatccATGGCTTAGGCTTTATTCGAT	*Bam*HI	pCAMBIA-1300-GN
MYB-pro_R	TTggtaccTGCTGTCTACTAACCTTTGC	*Kpn*I	
**For CRISPR/Cas9 mutation of MYB108 and identification of mutants**			
MYB-SP1_F	GGCATTGTCTTCCTCGAGGGTCCA		pOs-sgRNA
MYB-SP1_R	AAACTGGACCCTCGAGGAAGACAA		
MYB-SP2_F	GGCACTCAAGCCTGACATAAAGCG		
MYB-SP2_R	AAACCGCTTTATGTCAGGCTTGAG		
MYB-SP3_F	GGCATAGCGCAGCATCTGCCGGGG		
MYB-SP3_R	AAACCCCCGGCAGATGCTGCGCTA		
Cas9-PCR_F	ACAAGGGCAGGGATTTCG		
Cas9-PCR_R	ACTGGTGGATGAGGGTGGC		
MYB-PCR_F	GATTCCTCCTCCCCACTCGC		
MYB-PCR_R	CATCTGCTGGTCCCATCTCA		

Table S3. Primers used for RT-qPCR analysis

Primers	Sequence(5' to 3')	Amplicon/bp
Actin_qRT_F	GAGTCTGGCCCATCCATTGT	60
Actin_qRT_R	AGCATTCTTGGGTCCGAAGA	
MYB108_qRT_F	GCTACGTATCCAGCTGAGCTTAGC	107
MYB108_qRT_R	CGCTTTAATGGAGAGACATCACA	
PHT1;1_qRT_F	CGCTTCCGTACGAGTGGTAGT	146
PHT1;1_qRT_R	GGTTCTTTCAAATCCAGGGAAA	
PHT1;2_qRT_F	GACGAGACCGCCCAAGAAG	74
PHT1;2_qRT_R	TTTTCAGTCACTCACGTCGAGAC	
PHT1;4_qRT_F	TATTGCGGCTTAGATTGCATTAG	72
PHT1;4_qRT_R	TCCAAATCAAATGGGCACTAAG	
PHT1;6_qRT_F	TATAACTGATCGATCGAGACCAGAG	76
PHT1;6_qRT_R	TGGATAGCCAGGCCAGTTATATATC	
PHT1;8_qRT_F	AGAAGGCAAAAGAAATGTGTGTTAAAT	114
PHT1;8_qRT_R	AAAATGTATTCGTGCCAAATTGCT	
PHT1;9_qRT_F	AGAAAAACATAGGCTTGTCATCCTTT	80
PHT1;9_qRT_R	AAAACCTAAGAAGCACTGTAAATAAATCC	
PHT1;10_qRT_F	ATGTCGCCCATCCTTCCA	63
PHT1;10_qRT_R	TCGCTTTCCGACGATGATC	
IPS1_qRT_F	TTGGCAATTATTCGGTGGAT	115
IPS1_qRT_R	ACCATTTCACCATCCTCTTTATG	
miR399j_qRT_F	GGAGCATGTGAAGTCTTTTGTAGC	
miR399j_qRT_R	GGCAACTCTCCTTTGGCAGA	
PHO2.1_qRT_F	CGAGGAGGCGGGACATG	308
PHO2.1_qRT_R	GATCGACAGGAAGGTGC	
CPS1_qRT_F	GCTGTTACTATGCTGCCAATTGC	69
CPS1_qRT_R	GCTCAAAAATCACTTCAGAAATGTG	
KS1_qRT_F	GGCTCTATTTTGTCGGGCAAT	66
KS1_qRT_R	GCTCGTTGCTGATATCCCTGAT	
KAO_qRT_F	CACCAAAGTTTCCGATGAACAC	64
KAO_qRT_R	CTGGCTGTTTTCGGTTCCA	
KO2_qRT_F	GCTCCACCCGCTTCATGT	67
KO2_qRT_R	GCTTCCAATGAATATAATGGTTCACA	
GA20-ox2_qRT_F	GCCGCCTTCCAATTTTGG	67
GA20-ox2_qRT_R	GTACGTTTTGCTTCTTCTTGCAATAT	
GA3-ox2_qRT_F	TGGAGGAGTTTCACAAGGAGATG	66
GA3-ox2_qRT_R	GCGCCCTCAAGAACAACCT	
GA2-ox3_qRT_F	AGCCAGGTTGGATGGATAGCTA	67
GA2-ox3_qRT_R	GAGCCCACCTCACCACACA	
